# Clinical characteristics and radiation therapy modality of younger patients with early-stage endometrial cancer, a multicenter study in China’s real world

**DOI:** 10.1186/s12885-024-12090-3

**Published:** 2024-03-20

**Authors:** Kun Zhang, Tiejun Wang, Zi Liu, Jianli He, Xiaoge Sun, Wei Zhong, Fengjv Zhao, Xiaomei Li, Sha Li, Hong Zhu, Zhanshu Ma, Ke Hu, Fuquan Zhang, Xiaorong Hou, Lichun Wei, Lijuan Zou

**Affiliations:** 1 Department of Radiation Oncology, Peking Union Medical College Hospital Chin ese Academy of Medical Sciences & Peking Union Medical College, No. 1 Shuaifuyuan Wangfujing Dongcheng District, Beijing, People’s Republic of China; 2 Department of Radiation Oncology, The second hospital Affiliated by Jilin University, Changchun, People’s Republic of China; 3 Department of Radiation Oncology, First Affiliated Hospital of Xi’an Jiaotong University, Xi’an, People’s Republic of China; 4 Department of Radiation Oncology, The General Hospital of Ningxia Medical University, Yinchuan, Ningxia People’s Republic of China; 5 Department of Radiation Oncology, The Affiliated Hospital of Inner Mongolia Medical University, Hohhot, Inner Mongolia People’s Republic of China; 6 Gynaecological Oncology Radiotherapy, The Affiliated Cancer Hospital of Xinjiang Medical University, Urumqi, People’s Republic of China; 7 Department of Radiation Oncology, Gansu Provincial Cancer Hospital, Lanzhou, Gansu People’s Republic of China; 8 Department of Radiation Oncology, Peking University First Hospital, Beijing, People’s Republic of China; 9 Department of Radiation Oncology, The 940th Hospital of Joint Logistics Support force of Chinese People’s Liberation Army, Lanzhou, Gansu People’s Republic of China; 10 Department of Radiation Oncology, Xiangya Hospital Central South University, Changsha, Hunan People’s Republic of China; 11 Department of Radiation Oncology, Affiliated Hospital of Chi feng University, Chifeng, Inner Mongolia People’s Republic of China; 12 Department of Radiation Oncology, Xijing Hospital, Air Force Medical University of PLA (the Fourth Military Medical University, Xi’an, People’s Republic of China; 13 Department of Radiation Oncology, The Second Hospital of Dalian Medical University, Dalian, People’s Republic of China

**Keywords:** Endometrial neoplasms, Young, Survival analysis, Radiotherapy, EBRT, VBT

## Abstract

**Background:**

Endometrial cancer is a prevalent gynecologic malignancy found in postmenopausal women. However, in the last two decades, the incidence of early-stage has doubled in women under 40 years old. This study aimed to investigate the clinical and pathological characteristics and adjuvant therapeutic modalities of both young and not -young patients with early-stage endometrial cancer in China’s real world.

**Methods:**

This retrospective study analyzed patients with early-stage endometrial cancer at 13 medical institutions in China from 1999 to 2015. The patients were divided into two groups: young (≤ 45 years old) and non-young (> 45 years old). Statistical comparisons were conducted between the two groups for clinical characteristics, pathological features, and survival. The study also identified factors that affect local recurrence-free survival (LRFS) using Cox proportional risk regression analysis. Propensity score matching (1:1) was used to compare the effects of local control between vaginal brachytherapy (VBT) alone and pelvic external beam radiotherapy (EBRT) ± VBT.

**Results:**

The study involved 1,280 patients, 150 of whom were 45 years old or younger. The young group exhibited a significantly higher proportion of stage II, low-risk, lower uterine segment infiltration (LUSI), and cervical invasion compared to the non-young group. Additionally, the young patients had significantly larger maximum tumor diameters. The young group also had a significantly higher five-year overall survival (OS) and a five-year LRFS. Age is an independent risk factor for LRFS. There was no significant difference in LRFS between young patients with intermediate- to high-risk early-stage endometrial cancer who received EBRT ± VBT and those who received VBT alone.

**Conclusions:**

In the present study, young patients had better characteristics than the non-young group, while they exhibited higher levels of aggressiveness in certain aspects. The LRFS and OS outcomes were better in young patients. Age is an independent risk factor for LRFS. Additionally, VBT alone may be a suitable option for patients under 45 years of age with intermediate- to high-risk early-stage endometrial cancer, as it reduces the risk of toxic reactions and future second cancers while maintaining similar local control as EBRT.

**Supplementary Information:**

The online version contains supplementary material available at 10.1186/s12885-024-12090-3.

## Background

Endometrial cancer is the most prevalent gynecologic malignancy and the sixth most common tumor in women worldwide, with 417,000 new cases diagnosed annually. It is frequently diagnosed in postmenopausal women, with a median onset age of 61 years [1, 2]. In the past 30 years, the incidence of new diagnoses has risen across all age groups. Particularly, among women under 40 years of age, the incidence of early-stage, low-grade endometrial cancer has doubled from 2.9 to 6.0 per 100,000 population between 2000 and 2017 [1, 3]. Previous studies have reported on the clinical characteristics of premenopausal endometrial cancer patients. The age cutoffs range from 40 to 50. These studies indicate that young patients are frequently diagnosed with stage I cancers and rarely exhibit lymph node or distant metastasis. The most frequent pathologic type for young patients is endometrioid carcinoma [4]. Also, tumors in young patients are typically highly differentiated and have less DMI [5, 6]. However, large samples of Asian populations were not included in these studies. Certain studies suggest that age independently affects cause-specific survival [7], while others indicate that age is not an independent influence factor for recurrence [8]. The influence of age on prognosis remains unclear, particularly among Asian populations.

Endometrial cancer is treated primarily through surgery. Adjuvant treatments, including radiation therapy, chemotherapy, and endocrine therapy, may also be necessary and are determined by risk stratification. The current risk classification is based on the integration of known prognostic factors including pathologic type, stage, grading, and lymph-vascular space invasion (LVSI), as published by the European Society for Medical Oncology (ESMO), European Society for Radiotherapy and Oncology (ESTRO), and European Society of Gynecological Oncology (ESGO) [9]. Radiation therapy comprises vaginal brachytherapy (VBT) and pelvic external beam radiotherapy (EBRT). While EBRT can decrease the risk of recurrence, it is associated with more adverse reactions, including urinary incontinence, diarrhea, fecal leakage, lower functional and pain scores, and ultimately a diminished quality of life, compared to VBT [10]. A study demonstrated that VBT can effectively reduce the risk of vaginal recurrence in intermediate- and high-risk patients with fewer gastrointestinal side effects compared to EBRT [11]. It is important to reduce toxicity, especially for younger patients due to their longer life expectancy. Therefore, it is necessary to determine if VBT can provide effective disease control like EBRT while minimizing radiation toxicity in this population. Meanwhile, survival in young patients has not significantly improved in the last 30 years [4]. This suggests the need for further exploration of treatment modalities for this patient group.

This study summarized the clinical characteristics of patients diagnosed and treated between 1999 and 2015 in multiple centers across China, and elucidated the difference in characteristics between young and non-young patients, using 45 years as a cutoff. Also, a detailed comparison of the survival was performed between the two groups. Additionally, factors that influence local recurrence and death were explored in all patients. Finally, a retrospective analysis was conducted to determine whether a more conservative radiotherapy modality could be used for young patients.

## Methods

### Collection of clinical information

The study retrospectively reviewed the medical records at 13 tertiary hospitals in China. Patients diagnosed with stage I or II endometrial cancer (EC) according to the 2009 International Federation of Gynecology and Obstetrics (FIGO) staging system between January 1999 and December 2015 were included. All patients underwent hysterectomy/bilateral salpingo-oophorectomy followed by adjuvant radiotherapy. Complete clinical, pathological, and follow-up information was collected, including age at diagnosis, type of surgery, type of pathology, grading, deep myometrial infiltration (DMI), LVSI, lower uterine segment invasion (LUSI), cervical interstitial infiltration, 2009 FIGO stage, grade of differentiation, and ESMO-ESGO-ESTRO risk classification. Patient radiotherapy data was obtained from treatment records. Patients with insufficient information were excluded. Postoperative adjuvant radiotherapy may include EBRT ± VBT (EBRT ± VBT), or VBT alone. The targeting area for EBRT includes the upper half of the vagina and the vaginal stump, as well as the para-uterine, presacral, obturator, external iliac, and internal iliac lymphatic drainage regions. External irradiation was performed through four-field box radiotherapy, three-dimensional conformal radiotherapy, or intensity-modulated radiotherapy. High-dose-rate VBT was delivered with a single- or multi-channel irradiator to the upper part of the vagina. Patients underwent regular follow-up after radiation treatment, with appointments scheduled every 3 to 6 months for the first 2 years, then every 6 to 12 months for the next 3 years, and then annually. The primary endpoint included death, cancer-specific death, distant metastasis, local stump recurrence, and pelvic lymph node recurrence.

### Data analysis

Data analysis was conducted utilizing SPSS 27.0. The patients were divided into two groups, young (≤ 45) and non-young (> 45). The t-test, chi-squared test, and Fisher’s exact test were used to compare the differences in clinical and pathologic characteristics between the two groups of patients. The chi-squared test and Fisher’s exact test were used to compare the incidence of primary endpoint events between the two groups during the follow-up period. Kaplan-Meier (K-M) survival analysis with a log-rank test was used to compare the outcome between the two groups. Univariate and multivariate Cox proportional hazards regression analyses were used to determine the independent factors affecting the prognosis of EC. Propensity-matched scores (1:1) were conducted among young patients at intermediate and above risk between patients who received VBT alone and those who received EBRT ± VBT, to adjust for potential differences in the characteristics between the 2 groups. Propensity scores were estimated by a multiple logistic regression for covariates including 2009 FIGO staging, MI, LVSI, poorly differentiated and undifferentiated or not. The K-M survival analysis was used to compare the survival between the two matched treatment groups.

## Results

### Patients and tumor characteristics

The study involved 1280 patients with early-stage EC diagnosed and treated in 13 medical centers across China between 1999 and 2015. 150 patients were 45 years old or younger with a mean age of 39.9 years, and 1130 patients were over 45 years old with a mean age of 58.4 years. The median follow-up time was 56 months (IQR: 38–83) for young patients and 53 (IQR: 38–78) months for non-young patients. There was no significant difference in follow-up time between the two groups (*p* = 0.60). In young patients, Stage II (*p* = 0.004), LUSI (*p* < 0.001), and cervical invasion (*p* = 0.006) were significantly more frequent than in non-young patients. Additionally, the maximum tumor diameter was significantly larger in young patients compared to non-young patients (*p* < 0.001). However, the young patients had a significantly lower proportion of DMI (*p* < 0.001) and a higher proportion of low- (*p* = 0.002) or intermediate-risk (*p* < 0.001) (Table 1). There were no significant discrepancies between the two groups concerning the type of pathology, high-risk status, surgical method, chemotherapy, radiotherapy modality, second primary tumor occurrence, and side effects.

**Table 1:** Comparison of clinical and pathological characteristics

Characteristic	All *n* = 1280	Young (age ≤ 45) *n* = 150	Non-young (age>45) *n* = 1130	p
Age	56.27 ± 9.00	39.93 ± 0.39	58.44 ± 0.21	< 0.001
Pathological type				
endometrioid	1183(92.4%)	142(94.7%)	1041(92.1%)	0.26
Non endometrioid	97(7.6%)	8(5.3%)	89(7.9%)	
2009FIGO staging				< 0.01
Ia	592(46.3%)	86(57.3%)	506(44.8%)	0.004
Ib	525(41.0%)	34(22.7%)	491(43.5%)	< 0.001
II	163(12.7%)	30(20.0%)	133(11.8%)	0.004
Risk classification				< 0.01
Low risk	359(28.0%)	58(38.7%)	301(26.7%)	0.002
Intermediate risk	354(27.7%)	21(14.0%)	333(29.5%)	< 0.001
High-intermediate risk	240(18.8%)	27(18.0%)	213(18.8%)	0.802
High risk	324(25.3%)	44(29.3%)	280(24.8%)	0.228
Differentiation				
G1: Well differentiated	419(32.7%)	63(42.0%)	356(31.5%)	0.98
G2: Moderately differentiated	537(42.0%)	49(32.7%)	488(43.2%)	
G3/4: Poorly/Undifferentiated	233(18.2%)	31(20.7%)	202(17.9%)	
Gx: Undetermined	91(7.1%)	7(4.7%)	84(7.4%)	
Deep Myometrial infiltration (≥ 1/2 or < 1/2)				
≥ 1/2	608(47.5%)	46(30.7%)	562(49.7%)	< 0.001
Unknown	8(0.6%)	2(1.3%)	6(0.6%)	
Lymphovascular space invasion (+/-)				
(+)	227(17.7%)	26(17.3%)	201(17.8%)	0.891
Lower uterine segment invasion (+/-)				
(+)	349(27.3%)	70(46.7%)	279(24.7%)	< 0.001
Unknown	1(0.08%)	0	1(0.09%)	
Cervical invasion				
(-)	1024(80.0%)	105(70.0%)	919(81.3%)	0.006
Interstitial infiltration	163(12.7%)	30(20.0%)	133(11.8%)	
Gland infiltration	92(7.2%)	14(9.3%)	78(6.9%)	
Unknown	1(0.08%)	1(0.7%)	0	
Maximum diameter of tumor	3.40 ± 1.89	4.18 ± 2.22	3.27 ± 1.81	< 0.001
Unknown	455(35.5%)	43(28.7%)	412(36.5%)	
Surgery type (comprehensive or partial)				
Comprehensive surgical staging	887(69.2%)	115(76.7%)	772(68.3%)	0.071
Unknown	38(3.0%)	2(1.3%)	36(3.2%)	
Adjuvant therapy (chemoradiotherapy/radiotherapy)				
Chemoradiotherapy	253(19.8%)	35(23.3%)	218(19.3%)	0.49
Unknown	83(6.5%)	10(6.7%)	73(6.5%)	
Radiotherapy modality				
VBT	627(48.9%)	77(51.3%)	550(48.7%)	0.77
EBRT	155(12.1%)	16(10.7%)	139(12.3%)	
VBT + EBRT	498(38.9%)	57(38.0%)	441(39.0%)	
With second **primary tumor**	42(3.3%)	4(2.7%)	38(3.4%)	0.81
Acute side effects	660(51.6%)	68(45.3%)	592(52.4%)	0.104
Lower gastrointestinal	496(38.8%)	50(33.3%)	446(39.5%)	0.154
Upper gastrointestinal	282(22.0%)	35(23.3%)	247(21.9%)	0.898
Urinary system	137(10.7%)	13(8.7%)	124(11.0%)	0.320
Hematologic system	295(23.0%)	34(22.7%)	261(23.1%)	0.801
Late side effects	318(28.2%)	29(22.1%)	289(29%)	0.100
Gastrointestinal	153(13.6%)	10(7.7%)	143(14.4%)	0.056
Urinary system	72(6.4%)	3(2.3%)	69(6.9%)	0.239
Hematologic system	61(5.4%)	9(6.9%)	52(5.2%)	0.431
Lower limb edema	98(8.7%)	7(5.4%)	91(9.1%)	0.455

### Survival analysis

**Fig. 1:**
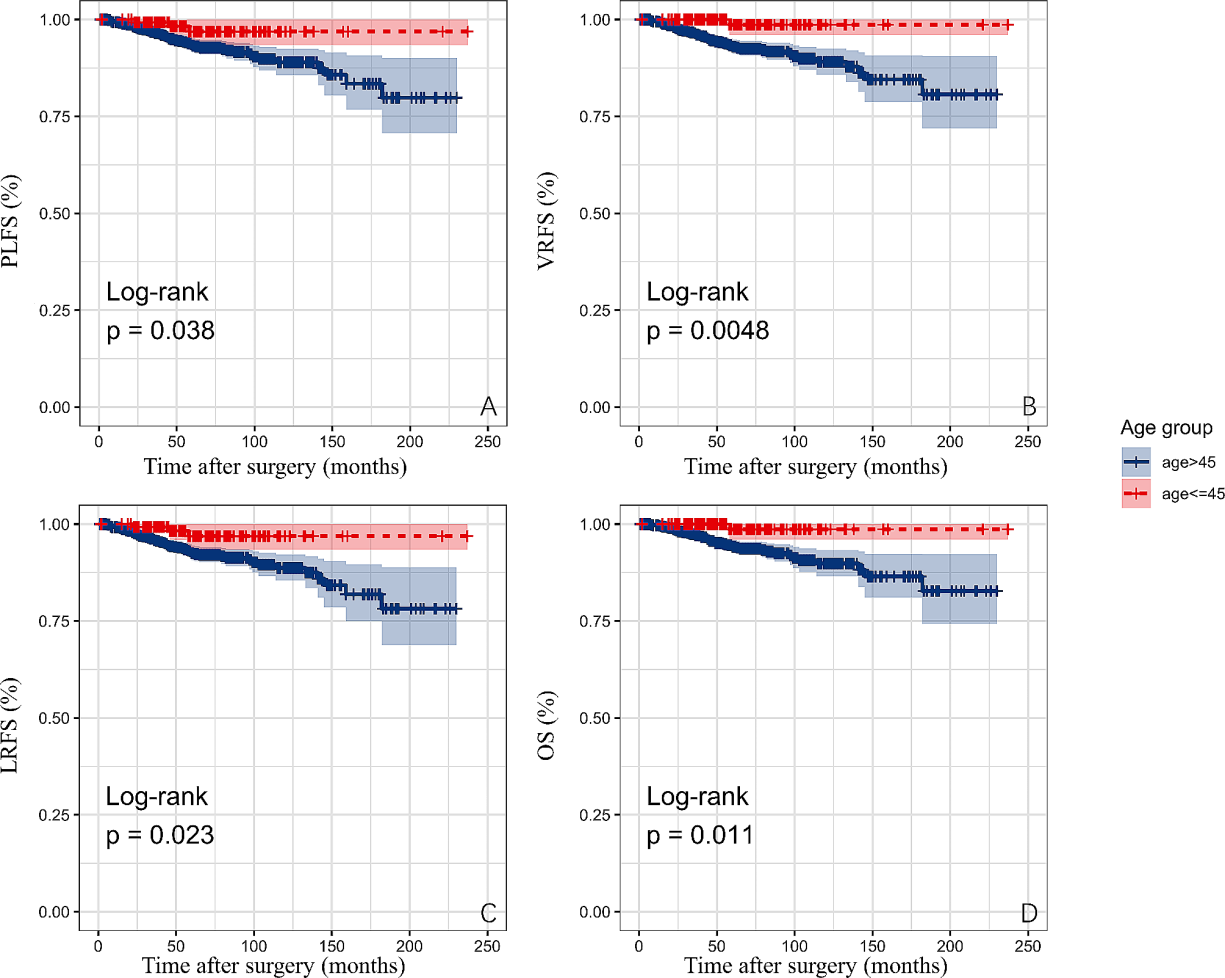
K-M survival analysis of young and non-young patients. PLFS, pelvic lymph node recurrence-free survival, LRFS, local recurrence-free survival, VRFS, vaginal stump recurrence-free survival, OS, overall survival

During the follow-up period, there were significant disparities between the two groups in the frequency of death (*p* = 0.012), vaginal stump recurrence or death (*p* = 0.005), pelvic lymph node recurrence or death (*p* = 0.04), and local recurrence or death (*p* = 0.019), which includes both stump and pelvic lymph node recurrence as well as death (Table 2). There was a significant difference in the 5-year overall survival (OS) between the two groups (young = 98.6%, non-young = 94.2%, log-rank test, *p* = 0.011). The 5-year local recurrence-free survival (LRFS) also showed a significant difference (young = 96.9%, non-young = 92.6%, log-rank test, *p* = 0.023). Additionally, young patients had significantly better pelvic lymph node recurrence-free survival (PLFS) (log-rank test, *p* = 0.038) and vaginal stump recurrence-free survival (VRFS) (log-rank test, *p* = 0.0048) compared to non-young patients (Fig. 1).

**Table 2:** Frequency of endpoint events

	All *n* = 1280	Young *n* = 150	Non-young *n* = 1130	p
Death	61	1	60	**0.012**
Treatment failure or death	108	7	101	0.077
Cancer-specific death	40	1	39	0.077
Distant metastasis or death	101	6	95	0.66
Vaginal stump recurrence or death	72	1	71	**0.005**
Pelvic lymph node recurrence or death	72	3	69	**0.04**
Local recurrence or death	79	3	76	**0.019**

The study then examined the factors influencing LRFS in all 1,280 patients. The results of the univariate analysis showed that age, differentiation, risk classification, and endometrioid carcinomas influenced LRFS. The multifactorial analysis showed that age was the only independent risk factor for LRFS (*p* < 0.001) (Table 3).

**Table 3:** Factors that affect the LRFS (Cox regression model)

	Univariate		Multivariate	
	HR (95%CI)	*p*	HR (95%CI)	*p*
Age	1.063(1.036–1.09)	**< 0.001**	1.060(1.033–1.088)	**< 0.001**
Endometroid (+/-)	0.479(0.246–0.932)	**0.03**	0.792(0.302–2.077)	0.635
Comprehensive surgery staging (+/-)	0.661(0.418–1.046)	0.077		
Maximum diameter of tumor	1.098(0.967–1.245)	0.148		
High-intermediate and high risk (+/-)	1.877(1.201–2.934)	**0.006**	1.314(0.672–2.573)	0.425
Poor differentiate and undifferentiated (+/-)	2.315(1.441–3.719)	**< 0.001**	1.852(0.907–3.708)	0.090
Myometrial infiltration ≧ 1/2(+/-)	1.290(0.824–2.021)	0.265		
LVSI (+/-)	1.249(0.711–2.196)	0.439		
LUSI (+/-)	1.276(0.798–2.041)	0.309		
Cervix invasion (+/-)	1.142(0.636–2.048)	0.657		
Radiotherapy modality (EBRT + VBT or EBRT/VBT)	1.108(0.708–1.733)	0.655		
Chemotherapy (+/-)	0.688(0.410–1.157)	0.158		

### Radiotherapy modality for young patients aged ≤ 45, intermediate- to high-risk

**Fig. 2:**
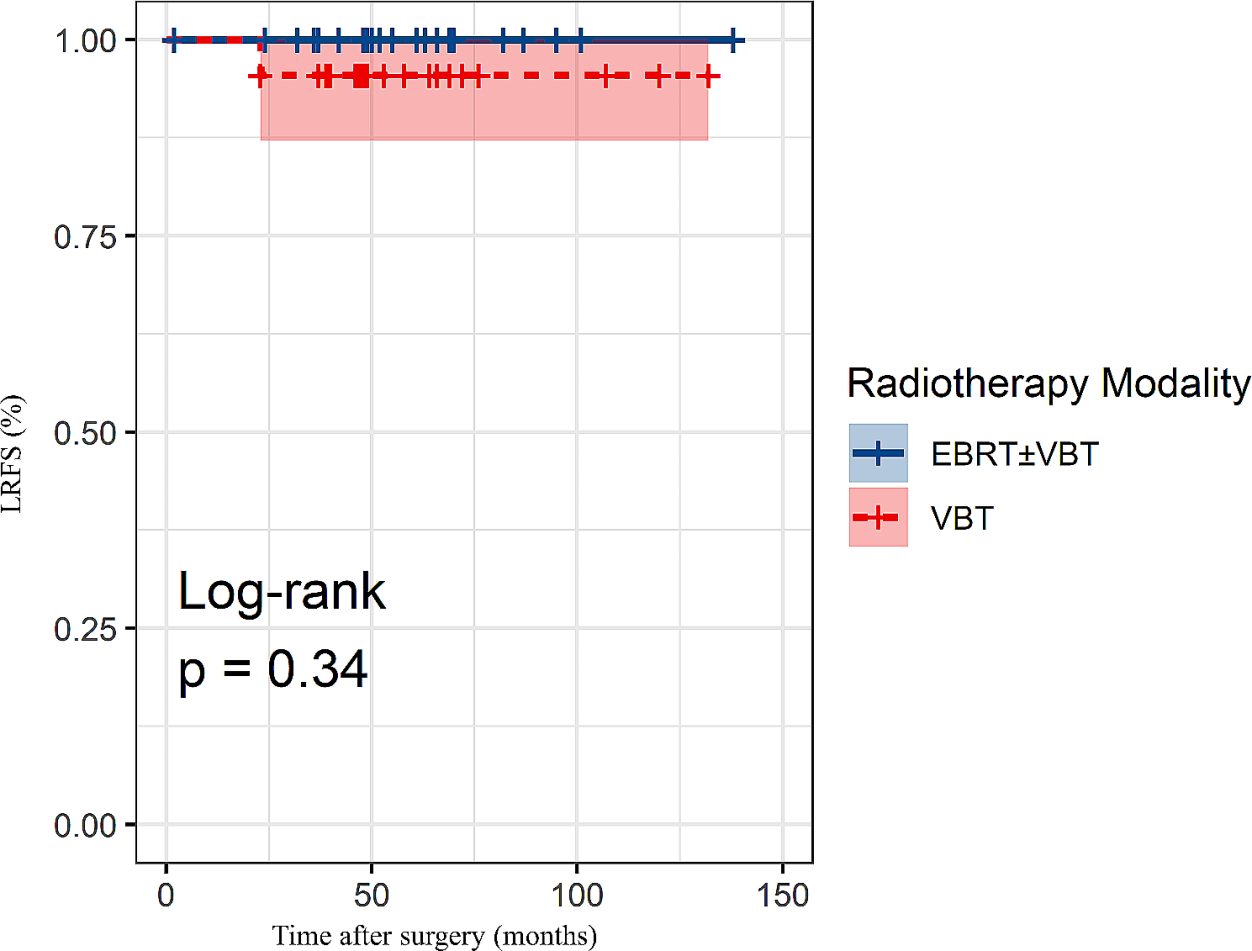
K-M survival analysis for LRFS after matching

To compare LRFS between patients who received EBRT ± VBT and those who received VBT alone, the propensity score matching method was used in intermediate- to high-risk patients younger than 45 years. Twenty-two pairs of patients were matched with no significant differences in their clinical characteristics (Table 4). K-M analysis revealed no significant disparity in LRFS between the two matched groups (log-rank test, *p* = 0.34) (Fig. 2), suggesting that a more conservative approach of VBT alone may be a viable treatment option for young patients with early-stage EC at intermediate- to high risk.

**Table 4:** Patient’s characteristics after matching

	EBRT ± VBT(*n* = 22)	VBT(*n* = 22)	*p*
Age	40.5 ± 3.66	41.77 ± 3.38	0.238
Endometroid carcer	20	22	0.488
2009 FIGO stage I	20	20	1
Poorly/undifferentiated	10	10	1
Myometrium infiltration ≥ 1/2	10	10	1
LVSI	4	4	1
LUSI	10	6	0.21
Cervix invasion (+)	6	2	0.118
Comprehensive staging surgery	15	17	0.337
Combined chemotherapy	7	3	0.284

## Discussion

Although EC typically occurs in postmenopausal women, recent epidemiological studies have shown an increasing proportion of young premenopausal patients. In particular, the incidence of low-grade EC has doubled in the 30–39 years age group [3]. According to data from the Metropolitan Detroit District Cancer Surveillance System from 1988 to 2007, patients under the age of 40 represent only 2.3% of all stage I and II patients [5]. As the population of this uncommon group grows, it becomes increasingly important to clarify their distinct characteristics. Studies have also revealed differences in characteristics distribution across various ethnic groups [12]. In addition, there are currently no established clinical guidelines indicating whether the treatment regimen for this group of patients should differ from that of postmenopausal patients. This study aimed to fill the gap by analyzing 1280 patients with early-stage endometrial cancer from 13 medical centers across China. It further elucidated the difference in the clinical characteristics and survival between young and non-young patients, using 45 years of age as the cutoff. Then, the study also explored the factors affecting LRFS, which showed that age acted as an independent risk factor. It also provided evidence to support the use of VBT alone rather than in combination with EBRT for young patients at intermediate- to high-risk.

Previous studies have reported on the characteristics of younger patients, but the conclusions regarding specific characteristics are not entirely consistent. A single-center study showed patients aged 45 years or younger had a comparable distribution of tumor stage, histologic type, and LVSI to patients aged 45 years or older, and younger patients had a lower frequency of deep myometrial infiltration and a higher prevalence of lower grades [13]. Another single-center study of patients between 1989 and 1994 also found that there was no significant difference in tumor stage between patients aged 45 years or younger and those who were older [14]. In previous studies using different age cutoffs, patients under 40 years old also exhibited a higher prevalence of low-grade and lower rates of DMI, but higher rates of endometrioid adenocarcinomas, which all indicated a better prognosis [5, 15]. In Asian populations, a study conducted in Taiwan revealed a higher incidence of endometrioid carcinoma and lower rates of DMI and LVSI in patients under 50 years old [16]. The distinct clinical characteristics of younger patients could indicate the biological and genetic diversity of endometrial cancer across various age groups [5, 17]. Overall, the previous results showed consistency in certain features, including less DMI and lower grades. Although the results on pathological type varied, most studies showed a higher incidence of endometrioid adenocarcinomas in young patients. These features mostly indicate better outcomes in younger patients. However, a trend toward more frequent lymph node involvement was observed in younger women [13]. LUSI has also been reported to be more prevalent in patients aged 40 years or younger [18]. Apart from the above, the present study found young patients had more stage II EC, larger maximum tumor diameters, and no difference in tumor grade or histological type compared to non-young patients. In addition, there were more frequent instances of LUSI and cervical invasion. These findings suggest that tumor characteristics in young patients may not be as favorable as previously thought. These characteristics may negatively impact the prognosis, which requires further attention from researchers.

A study of 34 early-stage patients exhibited a 91% 5-year OS in patients under 40 years of age [19]. Whereas, the present study demonstrated a 5-year OS of 98.6% in patients under 45 years with early-stage EC, with only one fatality during the follow-up period. The enhancement in survival may result from recent advancements in timely diagnosis and treatment. Previous studies have suggested that young patients have a better OS compared to older patients. A study conducted on a Taiwanese population discovered that endometrial cancer patients under the age of 50 had significantly better progression-free survival (PFS) and OS than those over 50 years old [16]. However, studies have demonstrated no significant disparity in 5-year PFS or cancer-specific survival (CSS) between patients under 45 years of age and patients over 45 years of age [13]. Another study conducted between 1989 and 1994 found no difference in OS between patients over and under 45 years of age [14]. Differences in conclusions may arise from the time and the region it was conducted, and variations in local medical conditions. The present research indicates that early-stage, younger patients experience superior OS but no significant disparity in PFS between the two groups. Furthermore, our study examined disease recurrence. While both groups exhibited similar distant metastasis-free survival (DMFS), a disparity in local control was observed, manifested in LRFS, PRFS, and VRFS. This indicates the advantage of local control for younger patients may contribute to their improved overall survival.

The noticeable variations in local control between the two patient groups prompted an investigation into the factors impacting prognosis. Several previous studies have examined the effect of age as a categorical variable on the prognosis. But the results remain controversial. The age of over 65 is an independent impact factor of OS and CSS [20]. However, another study found that the age of 70 or older did not independently affect OS in early-stage endometrioid adenocarcinoma patients after adjusting for other adverse prognostic factors [21]. Regarding recurrence, a study concludes that age over 70 is not an independent factor for tumor recurrence [8]. While another study shows that age over 80 is an independent factor for recurrence [22]. One study discovered that after controlling other risk factors, age over 70 didn’t associate with PFS but was associated with worse CSS [23]. However, in that study, the number of patients under the age of 70 receiving chemotherapy after recurrence was three times higher than that of advanced-age patients, so the correlation of age and CSS might be explained by different treatment patterns for various age groups after recurrence. Similarly, Laura Haley et al. demonstrated that age over 70 did not significantly impact CSS after controlling for other variables, whereas it significantly influenced the 5-year OS [24]. Differences among these findings might arise from variations in their age cut-off, differences in patient characteristics among different studies, differences in treatment modalities employed in distinct age subgroups, and differences in the era in which the study was conducted. These confounding factors made it difficult to accurately demonstrate the effect of age on patient prognosis, which needs further investigation in subgroups. Age as a continuous variable was found to be an independent factor for CSS. The hazard ratio (HR) for CSS progressively increases with age [7]. In the present study, age as a continuous variation was found to independently influence LRFS in patients with early-stage EC, which further contributes to the understanding of age’s impact.

Further, the study retrospectively examined adjuvant radiotherapy for young early-stage EC patients at intermediate to high risk. The result indicated that EBRT ± VBT did not provide better local control than VBT alone. The result is also supported by previous studies. In patients at intermediate to high risk, the EBRT ± VBT did not demonstrate a superior survival effect compared to VBT alone [25]. For grade 1 or 2 with DMI and grade 3 without DMI, VBT was as effective as EBRT in preventing vaginal recurrence [26]. Although the 15-year outcome of the PORTEC-1 study showed that EBRT had advantages for local control in stage I, low and intermediate-risk endometrial cancer patients, it did not confer any significant benefit in OS. Additionally, EBRT increases the risk of secondary malignancies, making it a wise choice to avoid EBRT [27]. Another long-term study also indicated no survival benefit from EBRT in early-stage EC patients. Additionally, women under the age of 60 who underwent EBRT experienced reduced survival and an increased risk of secondary cancers [28]. In patients with intermediate- and high-risk stage I and IIA, EBRT did not demonstrate an advantage over VBT in terms of OS and recurrence control. However, EBRT was associated with significantly more gastrointestinal side effects than VBT, and 10-year long-term results also showed favorable outcomes of VBT [11, 29]. However, there is a paucity of research on adjuvant radiotherapy modalities for premenopausal women. Based on the present and previous studies, VBT alone without EBRT is appropriate for patients aged under 45 years with early-stage intermediate- to high-risk. VBT provides an acceptable local control effect while avoiding more adverse effects and lowering the risk of future secondary malignancies. To further clarify the potential benefits of VBT alone for younger patients with early-stage EC, it is imperative to conduct prospective studies on adjuvant treatment modalities in the future.

The strength of the study lies in the enrollment of a large number of patients with early-stage endometrial cancer from 13 medical centers across China, which reduces selection bias. The clinical and pathological characteristics were thoroughly documented. Additionally, the follow-up period was quite long. Also, the study retrospectively investigated the application of adjuvant radiotherapy in young patients, serving as a foundation for further clinical research. However, the study has some limitations. The diversity of patient characteristics and confounding factors make it challenging to conduct further detailed subgroup studies. Additionally, the molecular subtype was missing due to the limited availability of genetic testing at the time of treatment. In future studies, incorporating molecular typing and conducting prospective studies can further strengthen the evidence base for optimal personalized radiotherapy. Maintaining the survival advantage while minimizing the potential for toxic reactions and finally improving the quality of long-term survival is the direction of modern radiotherapy.

## Conclusion

Our study elucidated distinct clinical and pathological features in young patients with early-stage EC, as compared to the typical postmenopausal patient population. Young patients exhibited higher rates of stage II, LUSI, and cervical invasion, as well as larger maximum tumor diameter compared to non-young patients. DMI is less frequent in young patients. The prognosis for early-stage endometrial cancer is generally favorable. However, young patients have a better prognosis than non-young patients, as reflected both in LRFS and OS. Age was an independent risk factor for LRFS. These findings suggest that the optimal adjuvant therapy for the young population may differ from that for the general population. Additionally, for patients under the age of 45 with early-stage intermediate- to high-risk EC, VBT provides comparable local control to EBRT, while also reducing potential side effects. Using VBT alone may be an appropriate treatment option for these patients. In the future, patients can be offered treatment options tailored to their own needs and preferences, such as VBT treatments, which are more convenient for those who have difficulty traveling to the medical center.

### Electronic supplementary material

Below is the link to the electronic supplementary material.

Supplementary Material 1

## Data Availability

The datasets used and analyzed during the current study are available from the corresponding author upon reasonable request.

## Abbreviations

EC: Endometrial cancer
LRFS: Local recurrence-free survival
OS: Overall survival
PLFS: Pelvic lymph node recurrence-free survival
VRFS: Vaginal stump recurrence-free survival
CSS: Cancer-specific survival
VBT: Vaginal brachytherapy
EBRT: External beam radiotherapy
EBRT ± VBT: External beam radiotherapy with or without vaginal brachytherapy
LUSI: Lower uterine segment infiltration
LVSI: Lympho-vascular space invasion
DMI: Deep myometrial infiltration
K-M: Kaplan-Meier
ESMO: European Society for Medical Oncology
ESTRO: European Society for Radiotherapy and Oncology
ESGO: European Society of Gynecological Oncology
FIGO: International Federation of Gynecology and Obstetrics
