# Supplementary material for: Clinical characteristics and radiation therapy modality of younger patients with early-stage endometrial cancer, a multicenter study in China’s real world
Source: BMC Cancer. 2024 Mar 20;24:360. doi: 10.1186/s12885-024-12090-3 (PMC10956334; doi:10.1186/s12885-024-12090-3)
Supplement: Supplementary file 1 — Supplementary Material 1 [file 12885_2024_12090_MOESM1_ESM.docx]

**Information on side effects**

We collected information on acute and late-phase side effects related to radiotherapy. Acute-phase side effects comprise upper gastrointestinal, lower gastrointestinal, urinary, and hematologic symptoms. Late-phase side effects comprise gastrointestinal, urinary, and hematologic symptoms and lower extremity edema. Of the patients who experienced acute side effects, only six non-young patients had grade 3 gastrointestinal reactions. Twenty non-young and three young patients developed grade 3 hematologic side effects, while three non-young and one young patient developed grade 4 hematologic side effects. Among the late side effects, only one non-young patient had a grade 3 urinary system reaction. Two non-young patients had grade 3 gastrointestinal side effects, and three non-young patients had grade 3 and 4 hematologic side effects. All patients who developed lower extremity edema were grades 1 or 2. There was no significant difference in the incidence of side effects between the two groups. The information on side effects has been added to Table 1.
